# Novel Surrogate Neutralizing Assay Supports Parvovirus B19 Vaccine Development for Children with Sickle Cell Disease

**DOI:** 10.3390/vaccines9080860

**Published:** 2021-08-04

**Authors:** Rhiannon R. Penkert, Sumana Chandramouli, Philip R. Dormitzer, Ethan C. Settembre, Robert E. Sealy, Susan Wong, Neal S. Young, Yilun Sun, Li Tang, Alyssa Cotton, Jola Dowdy, Randall T. Hayden, Jane S. Hankins, Julia L. Hurwitz

**Affiliations:** 1 Department of Infectious Diseases, St. Jude Children’s Research Hospital, Memphis, TN 38105, USA; rpenkert@uoregon.edu (R.R.P.); bob.sealy@stjude.org (R.E.S.); 2 Novartis Vaccines and Diagnostics, Cambridge, MA 02139, USA; sumana.chandramouli@gmail.com (S.C.); philip.dormitzer@pfizer.com (P.R.D.); ethan.settembre@seqirus.com (E.C.S.); 3 Hematology Branch, National Heart, Lung and Blood Institute, Bethesda, MD 20892, USA; wongsu@nhlbi.nih.gov (S.W.); youngns@nhlbi.nih.gov (N.S.Y.); 4 Department of Biostatistics, St. Jude Children’s Research Hospital, Memphis, TN 38105, USA; yilun.sun@stjude.org (Y.S.); li.tang@stjude.org (L.T.); 5 Department of Hematology, St. Jude Children’s Research Hospital, Memphis, TN 38105, USA; alyssa.cotton@stjude.org (A.C.); jola.dowdy@stjude.org (J.D.); jane.hankins@stjude.org (J.S.H.); 6 Department of Pathology, St. Jude Children’s Research Hospital, Memphis, TN 38105, USA; randall.hayden@stjude.org; 7 Department of Microbiology, Immunology and Biochemistry, University of Tennessee Health Science Center, Memphis, TN 38163, USA

**Keywords:** parvovirus B19, VP1u, neutralization assay, aplastic crisis, sickle cell disease, vaccine, enzyme-linked immunosorbent assay

## Abstract

Children with sickle cell disease (SCD) suffer life-threatening transient aplastic crisis (TAC) when infected with parvovirus B19. In utero, infection of healthy fetuses may result in anemia, hydrops, and death. Unfortunately, although promising vaccine candidates exist, no product has yet been licensed. One barrier to vaccine development has been the lack of a cost-effective, standardized parvovirus B19 neutralization assay. To fill this void, we evaluated the unique region of VP1 (VP1u), which contains prominent targets of neutralizing antibodies. We discovered an antigenic cross-reactivity between VP1 and VP2 that, at first, thwarted the development of a surrogate neutralization assay. We overcame the cross-reactivity by designing a mutated VP1u (VP1uAT) fragment. A new VP1uAT ELISA yielded results well correlated with neutralization (Spearman’s correlation coefficient = 0.581; *p* = 0.001), superior to results from a standard clinical diagnostic ELISA or an ELISA with virus-like particles. Virus-specific antibodies from children with TAC, measured by the VP1uAT and neutralization assays, but not other assays, gradually increased from days 0 to 120 post-hospitalization. We propose that this novel and technically simple VP1uAT ELISA might now serve as a surrogate for the neutralization assay to support rapid development of a parvovirus B19 vaccine.

## 1. Introduction

Parvovirus B19 usually does not cause serious disease in healthy individuals, but in patients with sickle cell disease (SCD), it can cause transient aplastic crisis (TAC), which can lead to permanent organ damage and death [1,2,3,4,5,6,7]. In the general population, parvovirus B19 also threatens fetal health and viability when infections occur during pregnancy.

**Figure 1:**
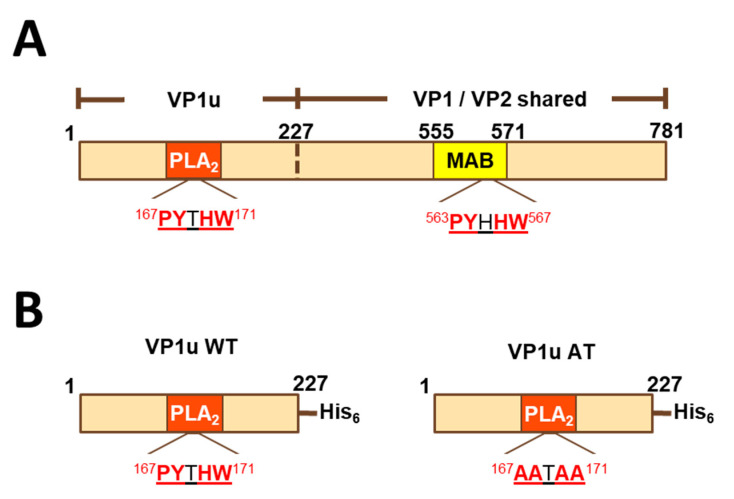
Linear diagrams of VP1 and its fragments. (**A**) Full-length VP1 sequence. VP1 and VP2 differ in that VP1 has an N-terminal extension (VP1u). Additionally, the sequence similarity of a 5-residue motif found in VP1u and VP2 is shown. (**B**) Histidine-tagged peptides VP1uWT and VP1uAT. MAB-MAB8293 epitope (residues 555–571 of VP1); PLA_2_-phospholipase A_2_-like domain; VP1u-VP1 unique region.

Currently, there is no licensed vaccine for parvovirus B19, although there are promising new candidates [8,9]. One promising vaccine candidate is a virus-like particle (VLP) composed of two proteins, VP1 and VP2 (VP1/VP2 VLPs). These two proteins are identical in most of their length (corresponding to residues 228–781 of VP1), but VP1 has an additional N-terminal extension (residues 1–227), termed the VP1 unique region (VP1u), an immunodominant peptide for neutralizing antibodies (Figure 1A) [10,11,12,13,14,15]. VP1u has phospholipase A_2_ (PLA_2_)-like activity, cleaving phospholipids into lysophospholipid (which can lyse membranes) and arachidonic acid (a precursor of inflammatory mediators). VP2 alone will assemble into VLPs [16], but is not effective at eliciting neutralizing antibodies [8,17]. VP1/VP2 VLPs better represent the parvovirus B19 virion compared to VP2 VLPs and elicit neutralizing antibodies, often directed against VP1u [10,11,12,13,14,15,17].

A VLP vaccine candidate was previously produced by co-infection of insect cells with two recombinant baculoviruses, expressing VP1 and VP2, respectively. The particles were not pure, had intact phospholipase A2 activity, and had variable VP1:VP2 ratios. In some immunized subjects, rashes appeared near the injection site [18]. Hypotheses for the cause of the reactogenicity focused on VLP impurities and PLA_2_ activity.

Next-generation VLPs were produced in stable yeast cell lines, which expressed VP1 and VP2 from a bicistronic plasmid [8]. This strategy ensured a fixed VP1:VP2 ratio. One cell line produced wild type, enzymatically active VLPs; another produced VLPs termed VP1mut/VP2 VLPs. The latter contained the full-length VP1 and VP2 proteins, but with an H153A mutation in the VP1u fragment of VP1 to abrogate PLA_2_ activity. The VLPs had indistinguishable physical characteristics, were highly pure, and elicited equivalent amounts of neutralizing antibodies in preclinical studies.

An outstanding barrier to the advancement of parvovirus B19 vaccine candidates has concerned the neutralization assay. In culture, parvovirus B19 only infects human erythroid progenitor cells (EPCs); cytotoxicity for these progenitors abrogates erythroid colony formation in vitro. Cell substrates used for parvovirus B19 neutralization assays include the UT7/Epo-S1 cell line, EPCs generated ex vivo from human hematopoietic stem cells, and EPCs differentiated and expanded from human peripheral blood mononuclear cells (PBMCs) [18,19]. The read out of the assay is detection of parvovirus B19 RNA transcripts by quantitative reverse-transcription polymerase chain reaction. These assays are technically challenging, time-consuming, low-throughput, expensive, and difficult to standardize, making them impractical for use in large scale clinical trials [19,20].

Because the VP1u peptide is an immunodominant neutralizing determinant, we sought to develop a VP1u peptide-based assay as a surrogate for neutralization. However, as described here, we discovered antigenic cross-reactivity between VP1 and VP2. The surprising result was that VP2-specific antibodies, which failed to neutralize parvovirus, bound to VP1u peptides. We redesigned the assay by introducing mutations into the VP1u peptide (alanine mutations introduced at residues Pro167, Tyr168, His170 and Trp171 to create VP1uAT) to abrogate antigenic cross-reactivity, and we compared the modified assay (VP1uAT ELISA) to other parvovirus B19 serological assays using sera from children with SCD hospitalized with parvovirus B19-induced TAC (iSCREEN protocol, ClinicalTrials.gov Identifier: NCT02261480) [21].

## 2. Methods

### 2.1. Expression and Purification of VP1u Peptides

Plasmids encoding VP1uWT (amino acids 1–227 of VP1) or mutant VP1u containing alanine mutations at residues Pro167, Tyr168, His170 and Trp171 (“VP1uAT”) were transformed into BL21 Star™ (DE3) chemically competent *E. coli* (Invitrogen, Waltham, MA, USA). Protein expression was induced using IPTG (Sigma-Aldrich, St. Louis, MO, USA) for 4 h at 37 °C. Cells were pelleted, resuspended and sonicated in buffer containing 25 mM Tris pH 7.5, 250 mM NaCl, 2.5 mM MgCl_2_, 20 mM imidazole, 10% glycerol, 0.2 mg/L lysozyme (Sigma-Aldrich), and 1 EDTA-free protease inhibitor cocktail tablet (Roche). Proteins were purified over a Ni-NTA column (Qiagen, Germantown, MD, USA) using a linear gradient of 20–500 mM imidazole in 25 mM Tris pH 7.5, 250 mM NaCl. A final step of size exclusion chromatography was performed using a Superdex 200 column equilibrated in 25 mM Tris pH 7.5 and 150 mM NaCl.

### 2.2. Western Blots of VP1u Peptides

For immunoblots, 0.5 μg of VP1uWT or VP1uAT were loaded onto SDS-PAGE gels, transferred to nitrocellulose membranes (Invitrogen, Waltham, MA, USA) and blocked with Odyssey blocking buffer (Licor, Lincoln, NE, USA) for 30 min, followed by incubation with primary antibody MAB8293 at 1 μg/mL and secondary antibody IRDye^®^ 800CW Goat anti-Mouse IgG (Licor) for 30 min each. Blots were washed between incubations 3× with PBS containing 0.5% Tween-20 and scanned using the Licor Odyssey Infra-Red scanner. Assays were at RT.

### 2.3. Mouse Immunizations

Animal immunizations were performed and described previously [8]. BALB/c mice were immunized intramuscularly three times, at three week intervals with MF59^®^-adjuvanted VP1mut/VP2 or VP2 VLPs at 5 µg each. Day 84 sera from eight mice per group were pooled. Animal experiments were performed in accordance with IACUC-approved protocols.

### 2.4. Monoclonal Antibody

The monoclonal antibody MAB8293 (EMD Millipore, Burlington, MA, USA), clone R92F6, is specific for residues 328–344 of VP2 (residues 555–571 of VP1) [22,23].

### 2.5. Clinical Samples

Participants in the iSCREEN protocol (Clinical trials.gov NCT02261480) were twenty-four sequentially hospitalized children (<18 years of age). Each child had a diagnosis of SCD (of any sickle genotype) and had active symptoms and signs of parvovirus B19-induced TAC. These children were enrolled over an 18 month period. (Table 1). Parvovirus B19-induced TAC was defined as worsened anemia with insufficient compensatory reticulocytosis in the setting of a febrile illness (or history of recent fever), with a positive diagnostic test for the virus (either a positive ELISA showing virus-specific IgM and/or IgG or a positive PCR). Participants were enrolled at the onset of TAC symptoms, and their serum samples were obtained longitudinally on days 0, 7, 30, and 120. On Day 30, four of these children were not confirmed to have parvovirus B19 and were removed from the study, leaving 20 patients for testing. The 20 patients, including ten females and ten males, had a mean age of 8.1 years. Sickle genotypes in these patients were HbSS (*n* = 15), HbSC (*n* = 3), HbSβ^+^-thalassemia (*n* = 1), and HbSD (*n* = 1).

There were an additional 29 samples from participants with SCD who were not experiencing aplastic crisis. In this case, ten females and 19 males had a mean age of 10.9 years. Sickle cell genotypes were HbSS (*n* = 26), HbSβ^+^-thalassemia (*n* = 2), and HbSβ^0^-thalassemia (*n* = 1).

The St. Jude IRB approved these studies, and an informed consent was signed by all legal guardians prior to study procedures. Verbal assent was obtained from minors who were between the ages of seven and 13 years, and signed assent was obtained from minors between 14 and 17 years of age. The IRB of St. Jude Children’s Research Hospital, Memphis, TN reviewed and approved this study. Personal identifiers were removed from all samples prior to analyses.

### 2.6. VLP binding ELISA

#### 2.6.1. VLP Binding ELISA with Mouse Sera

As described previously [8], for binding IgG titer determination, ELISA plates were coated with 1 µg/mL VP1/VP2 VLPs and incubated with 5-fold serial dilutions of sera starting at 1:625. A horse radish peroxidase (HRP)-conjugated goat anti-mouse IgG was used as a secondary antibody; 3,3′,5,5′-tetramethylbenzidine (TMB) substrate was used for detection; and color development was stopped with 1 M phosphoric acid. Plates were read at 450 nm (OD_450_). Readings from wells with no serum were used to determine background and were subtracted from readings from wells with serum. The binding IgG titer was calculated as the serum dilution giving a net OD_450_ of 0.5 after blank subtraction.

#### 2.6.2. VLP Binding ELISA with Human Sera and Nasal Washes (NW)

A B19 VLP VP1/VP2 Co-Capsid product was purchased from Prospec (cat# prv-002-c). VLPs were produced in SF9 insect cells, contained VP1 (86 kD) and VP2 (61 kD), and were purified using proprietary chromatographic techniques. Ninety-six-well ELISA plates were coated with 0.1 μg/mL VLP antigen (100 μL/well) and incubated overnight at 4 °C. Plates were washed with Dulbecco’s phosphate-buffered saline (DPBS) and blocked with 1% BSA in PBS for 1–2 h at 37 °C or overnight at 4 °C. Sera (diluted 1:100 and then serially diluted 1:10) or NW (diluted 1:10 and then serially diluted 1:10) were added in replicate wells for 1 h at room temperature (RT). Plates were washed, and goat anti-human IgG heavy chain conjugated to horse radish peroxidase (HRP, SBA#2040-05) or goat anti-human IgA heavy chain-HRP (SBA#2050-05; diluted 1:2000) was added. Plates were incubated for 1 h at RT. Plates were washed and developed with TMB (KPL) for 5 min. Reactions were stopped with 1M phosphoric acid. Average OD_450_ readings from matched samples on uncoated plates were subtracted from test OD_450_ readings. Titers were determined using a non-linear regression program (GraphPad Prism, San Diego, CA, USA). The cut-off for positivity was an OD_450_ reading of 0.1.

### 2.7. VP1uWT and VP1uAT Binding ELISAs

#### 2.7.1. VP1uWT and VP1uAT Binding IgG ELISAs with Mouse Sera

ELISA plates (Nunc MaxiSorp 96-well) were coated with VP1uWT or VP1uAT peptides at 1 μg/mL in PBS at 0.1 mL per well overnight at 4 °C. Wells were blocked with SuperBlock (Pierce ThermoFisher Scientific, Waltham, MA, USA) for 1–2 h at 37 °C. The assay diluent was PBS containing 5% goat serum (GIBCO Invitrogen, Waltham, MA, USA) and 0.1% Tween 20. Serial dilutions were made with sera from mice immunized with VLPs. The monoclonal antibody MAB8293 was also used following serial dilution. Antibodies were incubated on plates for 2 h at 37 °C followed by addition of detection antibody (HRP conjugated goat anti-mouse IgG antibody, SouthernBiotech, Birmingham, AL, USA) for 1 h at 37 °C. After incubation with TMB substrate for 20 min at room temperature (RT) in the dark, 1 M phosphoric acid was added to the wells to stop the reactions. Washes between steps were with PBS, 0.1% Tween-20. Wells with no serum were included as blanks. Plates were read at 450 nm and fit to a 4-parameter curve (GraphPad Prism). The binding IgG titer was calculated as the serum dilution giving a net OD_450_ of 0.5 after blank subtraction.

#### 2.7.2. VP1uWT and VP1uAT Binding IgG ELISAs with Human Sera

Plates were coated with 0.1 μg/mL VP1uWT or VP1uAT antigens. Assays were then performed with the same methods described for the IgG VLP-based ELISA with human sera.

### 2.8. Diagnostic ELISA with Human Sera

A diagnostic test for antibodies recognizing parvovirus B19 was performed by Quest Diagnostics (Secaucus, NJ, USA) or ARUP Laboratories (Salt Lake City, UT, USA). Companies used a commercial kit (B19V-specific EIAs for B19V-specific IgM and IgG [Biotrin International, Dublin, Ireland]). These ELISAs targeted a baculovirus-expressed VP2 antigen.

### 2.9. Neutralization Assays

#### 2.9.1. Neutralization Assay with Mouse Sera

As described previously [8], neutralizing titers of mouse sera were measured using an RT-PCR-based method and CD36+/globoside+ erythroid progenitor cells (EPCs) derived from PBMCs. Five-fold serial dilutions of mouse sera starting at 1:20 were pre-incubated with virus and added to the cells for 48 h. RNA extracted from the cells was assayed by quantitative RT-PCR for viral RNA using multiplex analysis and normalized based on β-actin mRNA levels. The assay was performed in duplicate, and each neutralization titer was determined as an inhibitory dose 50 (ID_50_), the serum dilution that resulted in a 50% reduction in signal compared to a no serum control.

#### 2.9.2. Neutralization Assay with Human Sera

The neutralization assay was conducted as previously described [18,19]. Briefly, 10 µL of serially diluted (1:10, 1:100, and 1:1000) sera were pre-incubated for 1 h at RT with equal volumes of human serum containing 2 × 10^6^ genome equivalents of parvovirus B19 (strain V2, [24]). Then, the mixture was added to 2 × 10^4^/10 µL primary EPCs propagated as previously described [19], for 2 h in a 96-well plate, and the volume was adjusted to 100 µL. Cells were cultured for 3 days at 37 °C in 5% CO_2_. RNA was extracted from the culture using a TurboCapture ^®^96 mRNA kit (Qiagen) and converted to cDNA using a MMLV-RT kit (ThermoFisher Scientific, Waltham, MA, USA). A real time, quantitative, multiplex PCR assay was performed to detect the cDNA from the parvovirus B19 NS and human β-actin transcripts, using the PerfeCTa Multiplex qPCR Super Mix (Quanta Biosciences Inc., Gaithersburg, MD, USA). Probes were from Integrated DNA Technologies: NS-1354 F (5′-GGGCAGCATGTGTTAAAGTGGA-3′), NS-1453 R (5′-TGGCCATTGCCAAGTTTGT-3′), NS-1413 probe (5TYE665-TTATGGGCCGCCAAGTACAGGAAA-31AbRQsp), β-actin F (5′-GGCACCCAGCACAATGAAG-3′), β-actin R (5′-GCCGATCCACACGGAGTACT-3′) and β-Actin probe (5MAX550-TCAAGATCATTGCTCCTCCTGAGCGC-3IABlk_FQ). Virus genome copies were determined by extrapolating from a standard curve generated from known quantities of target. To validate the number of infectious particles in the stock human serum, 10-fold serial dilutions of virus stock V2 were used to infect EPCs after incubation with seronegative control serum. Neutralization titers were defined as the inverse of the highest serum dilution that reduced viral transcripts by 90%.

### 2.10. Statistics

Spearman’s correlation coefficients were calculated to evaluate the correlation between quantitative assay results. Cohen’s kappa coefficients were utilized to evaluate qualitative agreement among assays. The sensitivity and specificity of VP1u and VLP IgG assays were assessed, treating the neutralization assay as the gold standard. Statistical analyses were performed using SAS version 9.4 (SAS Institute, Cary, NA, USA) and GraphPad Prism, version 7.

## 3. Results

### 3.1. Binding of Parvovirus B19 VLPs, Neutralization of Parvovirus B19, and Binding of a VP1u Peptide by Serum Antibodies from VLP-Immunized Mice

**Figure 2:**
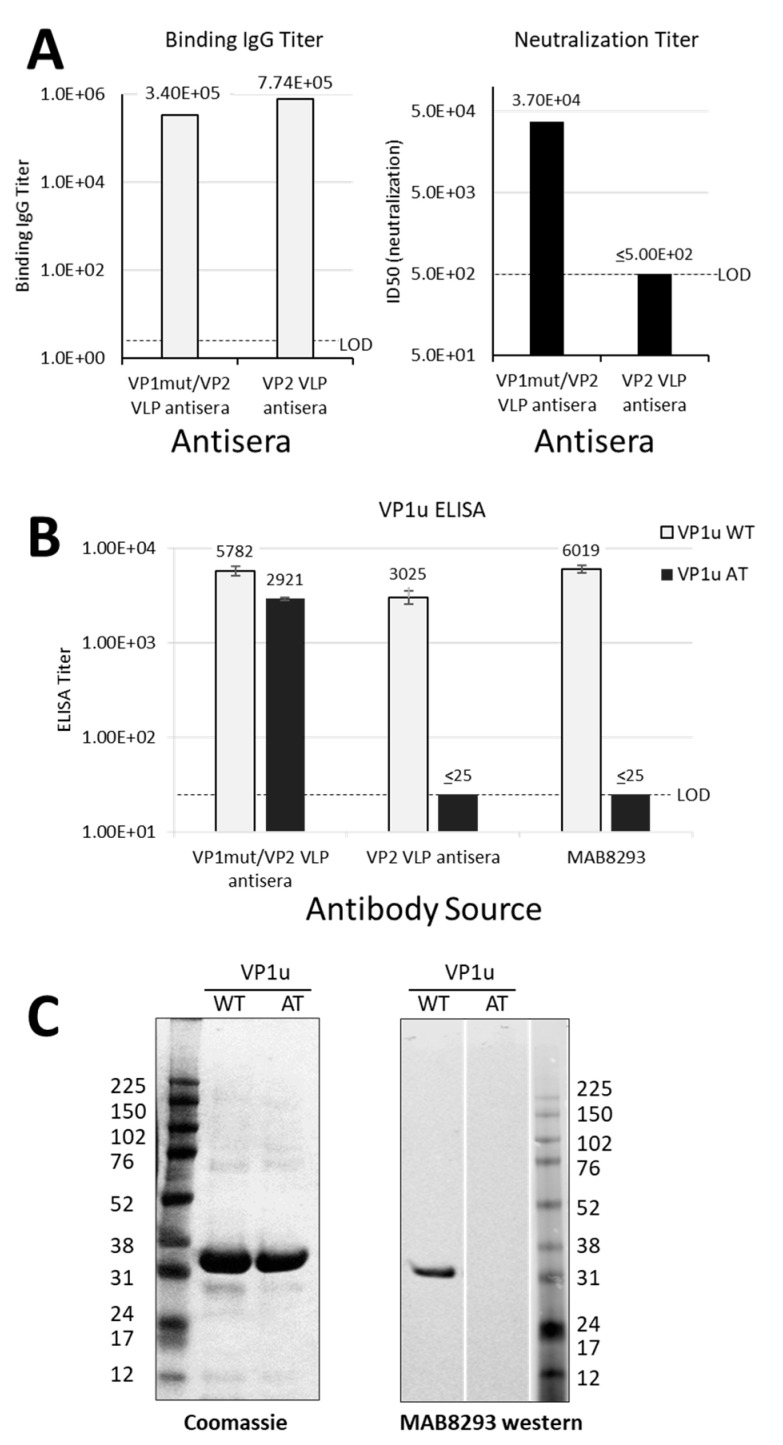
Differential antibody binding and function. (**A**) Results in A are graphed from data that were previously reported in table form by Chandramouli et.al. [8]. Pooled sera were from eight mice per group that were immunized either with VP1mut/VP2 VLP or VP2 VLP (see x-axes). These pooled sera were used in an ELISA with plates coated with VP1mut/VP2 VLPs (left). The lower limit of detection for this assay was determined using pre-immune sera (LOD, titer = 5). The same pooled sera were used in a neutralization assay with parvovirus B19 (right). The LOD of the neutralization assay was a titer of 500. (**B**) The same pooled mouse sera were newly tested in ELISAs with plates coated with peptides VP1uWT or VP1uAT (see legend). The monoclonal antibody mAb8293 was also tested. Means are shown with standard error bars. The LOD of these ELISAs was a titer of 25. (**C**) Purified VP1uWT and VP1uAT peptides were separated on SDS-PAGE and Coomassie-stained (left). Peptides were also tested by western blot, developed with monoclonal MAB8293 (right).

We previously reported a study of mice immunized with yeast-expressed parvovirus B19 VLPs, adjuvanted with MF59 [8]. One VLP, termed ‘VP1mut/VP2′ included both VP1 and VP2 and carried an H153A mutation in VP1, which was introduced to abrogate phospholipase A_2_ activity. Figure 1A shows the shared and unique sequences of VP1 and VP2. The second VLP was comprised of only VP2 (termed ‘VP2-only’). In our previous analyses, we found that both VLPs elicited antibodies that bound the VP1mut/VP2 VLP in an ELISA, but only the VP1mut/VP2 VLP elicited detectable neutralizing antibodies. Our previously described data are now graphed in Figure 2A [8].

Because VP1 and VP2 differ by the VP1u N-terminal extension (Figure 1A) and because VP1u is an important target of neutralizing antibodies [10,11,12,13,14,15], we hypothesized that antibody binding to VP1u would correlate with parvovirus B19 neutralization. To test this hypothesis, we expressed a histidine-tagged VP1u construct (residues 1–227 of VP1) as a soluble protein (VP1uWT) in *E. coli* (Figure 1B). VP1u WT, which had measurable phospholipase activity in vitro (data not shown), was produced with yields of approximately 20 mg/L, purified, and used to coat ELISA plates.

The mouse sera from our previous immunization study were then newly tested for binding in VP1u-based ELISAs [8]. As expected, the parvovirus B19 neutralizing mouse sera elicited by immunization with VP1mut/VP2 VLPs recognized VP1uWT by ELISA (Figure 2B). However, unexpectedly, the non-neutralizing sera elicited by mouse immunizations with VP2 VLPs also recognized VP1uWT.

As a negative control for the VP1uWT ELISA, we included a monoclonal antibody, MAB8293 (Millipore), which is directed against a VP2 epitope (VP2 residues 328–344, corresponding to VP1 residues 555–571) (Figure 1A). This antibody also recognized VP1uWT (Figure 2B), a result that was confirmed by western blot (Figure 2C, right panel). The antibody cross-reactivity between VP1uWT and VP2 was again unexpected. Furthermore, contrary to our hypothesis, the ability of sera to bind VP1uWT did not correlate well with parvovirus B19 neutralization.

### 3.2. Identification of a Shared Sequence Motif between VP1u and VP2

To explain antibody cross-reactivity between VP1uWT and VP2, we searched for an epitope shared by the two sequences. Indeed, amino acid sequence alignment showed that residues 167–171 of VP1uWT (PYTHW), and residues 336–340 of VP2 (PYHHW; corresponding to 563–567 of VP1; Figure 1A) shared four of the five residues. In VP1uWT, Y168 is the catalytic tyrosine of the PLA_2_ domain (Figure 1B). In VP2, the motif overlaps the MAB8293 epitope.

### 3.3. Abrogation of Antigenic Cross-Reactivity with VP2 by Mutation of VP1uWT

If antigenic similarity of the shared motif explained the recognition of VP1uWT by VP2-specific antibodies, mutating the motif in VP1uWT would be expected to eliminate cross-reactivity. We expressed a new VP1u construct (VP1uAT), in which the motif’s four conserved residues (P167, Y168, H170 and W171) were each mutated to alanine, leaving the non-conserved T169 unchanged (Figure 1B). Like VP1uWT, VP1uAT was soluble when expressed in *E. coli* and was purified with high yields (Figure 2C, left). The purification profiles of VP1uAT and VP1uWT were similar. Unlike VP1uWT, VP1uAT was not recognized by MAB8293, confirming the antigen cross-reactivity hypothesis (Figure 2B,C, right panel). When our mouse sera were tested in the VP1uAT ELISA, binding patterns matched patterns of neutralization (Figure 2A,B).

### 3.4. Antibody Responses in Children Suffering TAC

To confirm the correlation of antibody binding in the VP1uAT ELISA with parvovirus B19 neutralization, we used human sera from an iSCREEN study that included twenty children with SCD sampled longitudinally following hospitalization with TAC.

**Figure 3:**
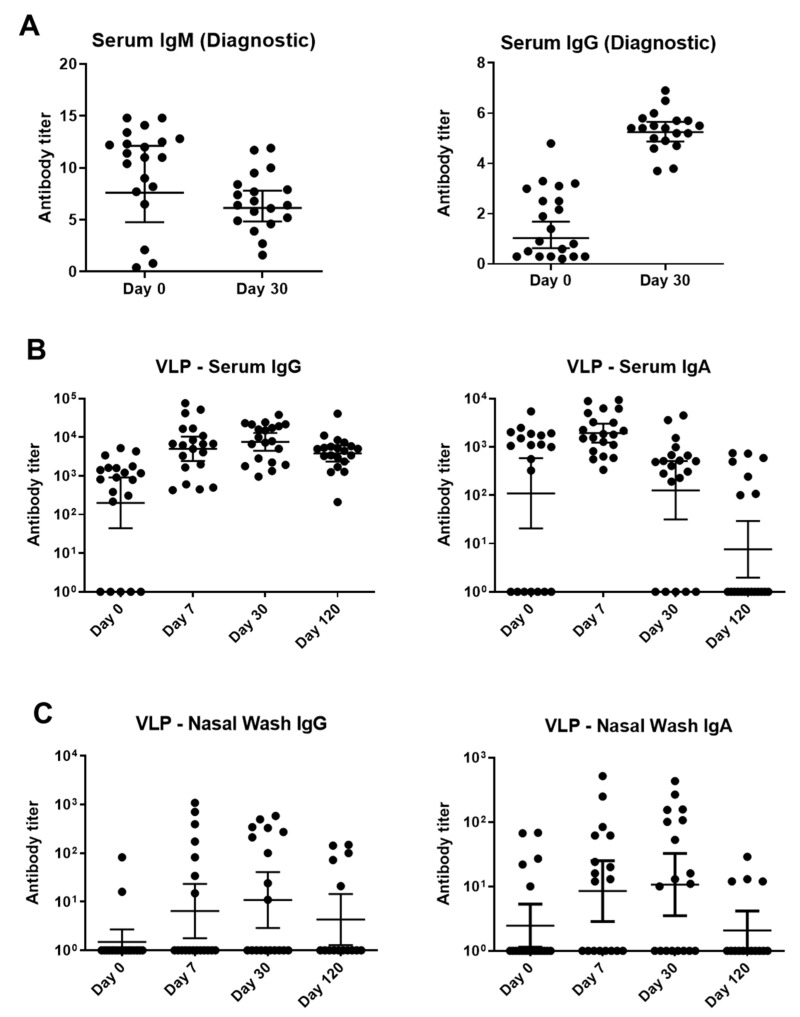
Binding antibody responses to parvovirus B19 infections among children with SCD and TAC. (**A**) A Biotrin kit-based ELISA with a baculovirus-vectored, insect cell-expressed parvovirus B19 VP2 antigen was performed at a commercial, CLIA-certified laboratory. Virus-specific IgM (left) and IgG (right) were detected. (**B**,**C**) Samples were tested against Prospec VP1/VP2 VLP in ELISAs. These VLPs were produced in SF9 insect cells. Sera (**B**) and nasal washes (**C**) from longitudinal patient samples were tested for IgG and IgA. Titers below detection in each assay were assigned a value of 10°. Geometric means are shown with 95% confidence intervals.

A semi-quantitative, commercially available, diagnostic ELISA was first used to identify IgM and IgG VP2-specific antibodies. As demonstrated in Figure 3A, VP2-specific IgM titers were generally higher on day 0 whereas IgG titers were higher on day 30.

A second ELISA used parvovirus B19 VLPs purchased from Prospec (containing VP1 and VP2, produced in SF9 insect cells) to measure IgG and IgA. This assay tested sera and NW from days 0, 7, 30, and 120 (Figure 3B,C). NW were examined because the respiratory tract is an important site of viral entry. VLP-specific IgG and IgA from sera and NW peaked early and began to wane by day 120 (Figure 3).

### 3.5. Functional Antibodies against Parvovirus B19 in Children with SCD and TAC

**Figure 4:**
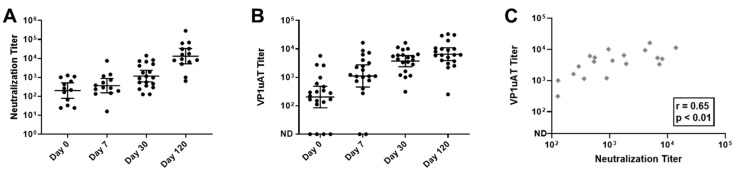
Kinetics of neutralization and VP1uAT ELISA responses to parvovirus B19 infections among children with SCD and TAC. Longitudinal serum samples from hospitalized patients were assayed. (**A**) Parvovirus B19 neutralization titers were defined as the inverse of the highest serum dilutions that reduced viral transcripts by 90%. (**B**) VP1uAT ELISA results. ND = antibodies not detected. Geometric means are shown with 95% confidence intervals. (**C**) VP1uAT and neutralization results on Day 30 are shown with Spearman’s correlation r and *p* values.

A neutralization assay was performed and samples from all 20 participants yielded positive scores. However, unlike the results from the VLP-based ELISA, the parvovirus B19 neutralizing response increased throughout the time course (Figure 4A).

IgG responses were also tested in the VP1uAT ELISA. As shown (Figure 4B,C), responses exhibited similar kinetics to those observed for neutralization. For many patients, the highest response was on Day 120. Thus, the VP1uAT ELISA and parvovirus B19 neutralization assays were well correlated.

To further compare VP1uAT and neutralization assays, an additional 29 samples from SCD patients without TAC at the time of sampling were tested. Data from all 49 samples were combined and showed an excellent qualitative agreement between the VP1uAT ELISA and the neutralization assay (kappa coefficient = 0.93, Table 2). By using the neutralization assay as a standard, the sensitivity and specificity of the VP1uAT assay were 97.6% and 100%, respectively (Table 2). A more modest agreement was observed between IgG that bound VP1/VP2 VLPs in ELISAs and neutralization (kappa coefficient = 0.63, Table 2). For 28 of the 49 samples (57%), neutralization titers scored above background and could be quantified by non-linear regression. For these samples, there was a strong positive correlation between the results of the VP1uAT ELISA and the neutralization assay (Spearman’s correlation coefficient = 0.581; *p*-value = 0.001). Again, a more modest correlation was observed between the IgG results from the VP1/VP2 VLP-based ELISA and the neutralization assay (Spearman’s correlation coefficient = 0.476; *p*-value = 0.011).

## 4. Discussion

Neutralization assays are fundamental tools for understanding immunity to viral infections and for developing vaccines. Unfortunately, the parvovirus B19 neutralization assay is variable, difficult to perform, and low-throughput. It requires training, equipment, and reagents that are not easily accessed by clinical laboratories. We therefore pursued the development of a practical surrogate assay.

The finding that detectable neutralizing activity can be elicited by immunization of mice with VLPs that contain VP1 and VP2 but not by VLPs that contain VP2 alone has been previously described [8,17]. Immunization with a recombinant fusion protein containing VP1u (which distinguishes VP1 from VP2) also elicits neutralizing antibodies [13]. However, VP1u is not the only target of parvovirus B19 neutralizing antibodies. From mice immunized with parvovirus B19 virions or synthetic VP2 peptides and from parvovirus B19-exposed humans, neutralizing antibodies have been obtained that map to VP2 on the basis of binding to synthetic or *E. coli*-expressed peptides, to denatured proteins on western blots, or to intact VLPs by immunoprecipitation [10,11,12,14,15,25]. Although some antibodies that map to parvovirus B19 VP2 can neutralize virus, the apparent dominance of antibodies recognizing VP1u in the neutralization response defines VP1u as a key antigenic target for vaccine development [10,11,12,13,14,15].

Our initial attempts to develop a surrogate for the neutralization assay were thwarted by unexpected antigenic cross-reactivity between a five-residue motif shared by VP1u and VP2 (Figure 1 and Figure 2). The cross-reactivity of this shared motif was best demonstrated by the binding pattern of monoclonal antibody MAB8293 (Millipore, clone R92F6), raised by immunization of mice with parvovirus B19 virions [10,25]. Mutating the shared motif between VP1u and VP2 abrogated cross-reactivity (Figure 2B,C). When we compared parvovirus B19 neutralization and the VP1uAT ELISA to two other parvovirus B19 serological assays with sera from children with SCD, the best correlation was between the VP1uAT ELISA and virus neutralization.

The study of children with TAC provided additional insights. The diagnostic ELISA with VP2 showed higher IgM responses on day 0 and higher IgG responses on day 30. The VLP-based ELISA showed IgG and IgA responses that often peaked on day 7. Both virus neutralization and the VP1uAT ELISA had distinctly different kinetics from others—responses continuously increased over time. Perhaps virus infection initially elicits antibodies with low neutralization potential and antibody affinity maturation is required for improved function.

In summary, new vaccine candidates are being developed to prevent parvovirus B19. One such candidate is a yeast-derived VLP vaccine that has proven effective in BALB/c mice and in a mouse model for SCD [9]. The VP1uAT ELISA may now serve as a scalable quantitative endpoint to support clinical trials with new vaccines.

## Figures and Tables

**Table 1:** Patient Characteristics.

iSCREEN ID	Sex	Age at Time of Enrollment (years)	Sickle Genotype
SC001	Male	6.8	HbSS
SC002	Male	5.5	HbSS
SC004	Female	7.2	HbSS
SC005	Male	5.8	HbSS
SC006	Female	8.5	HbSC
SC007	Female	6.7	HbSS
SC008	Male	6.1	HbSS
SC011	Male	14.3	HbSS
SC012	Female	9.8	HbSS
SC013	Male	4.5	HbSS
SC014	Female	7.2	HbSS
SC016	Male	10.6	HbSS
SC017	Female	6.2	HbSS
SC018	Female	11.4	HbSβ^+^ Thalassemia
SC019	Male	6.0	HbSD
SC020	Female	7.0	HbSS
SC021	Male	11.8	HbSS
SC022	Female	8.8	HbSC
SC023	Female	5.3	HbSS
SC024	Male	12.8	HbSC

**Table 2:** Kappa coefficient, sensitivity and specificity of VP1uAT ELISA and VLP IgG ELISA with the neutralization assay as the gold standard.

	Qualitative VP1u Results		Qualitative VLP IgG Results	
Qualitative Neutralization Results	Positive	Negative	Positive	Negative
Positive ^a^	40	1	36	5
Negative	0	8	1	7
Kappa coefficient (95% CI)	0.93 (0.79, 1.00)		0.63 (0.36, 0.89)	
Sensitivity (95% CI)	97.6% (87.1%, 99.9%)		87.8% (73.8%, 95.9%)	
Specificity (95% CI)	100%		87.5% (47.4%, 99.7%)	

^a^ Serum samples were obtained from patients 120 days after hospitalization for acute parvovirus B19 infection or from additional patients who were not experiencing aplastic crisis.

## Data Availability

Data requests may be made to authors.

## Abbreviations

NW	nasal wash
SCD	sickle cell disease
EPC	erythroid progenitor cells
ELISA	enzyme linked immunosorbent assay
LOD	limit of detection
TAC	transient aplastic crisis
IACUC	Institutional Animal Care and Use Committee
VLP	virus-like particle
VP1wt	A full-length VP1 protein with wildtype sequence
VP1mut	A full-length VP1 protein with an H153A mutation
VP1u	VP1 unique region
VP1uWT	VP1 unique region with wildtype sequence
VP1uAT	VP1 unique region with alanine mutations
